# Congruence of chloroplast- and nuclear-encoded DNA sequence variations used to assess species boundaries in the soil microalga *Heterococcus* (Stramenopiles, Xanthophyceae)

**DOI:** 10.1186/1471-2148-13-39

**Published:** 2013-02-13

**Authors:** Nataliya Rybalka, Matthias Wolf, Robert A Andersen, Thomas Friedl

**Affiliations:** 1 Experimental Phycology and Culture Collection of Algae (SAG), Georg August University Göttingen, Untere Karspüle 2a, Göttingen, 37073, Germany; 2 Plant Cell Physiology and Biotechnology, Botanical Institute, Christian Albrechts University of Kiel, Am Botanischen Garten 1-9, Kiel, 24118, Germany; 3 Department of Bioinformatics, Biocenter, University of Würzburg, Würzburg, 97074, Germany; 4 Friday Harbor Laboratories, University of Washington, Friday Harbor, WA, 98250, USA

**Keywords:** Soil algae, *Heterococcus*, Xanthophyceae, *psb*A/*rbc*L spacer, ITS2, Systematics, Molecular phylogeny, Species concept

## Abstract

**Background:**

*Heterococcus* is a microalgal genus of Xanthophyceae (Stramenopiles) that is common and widespread in soils, especially from cold regions. Species are characterized by extensively branched filaments produced when grown on agarized culture medium. Despite the large number of species described exclusively using light microscopic morphology, the assessment of species diversity is hampered by extensive morphological plasticity.

**Results:**

Two independent types of molecular data, the chloroplast-encoded *psb*A/*rbc*L spacer complemented by *rbc*L gene and the internal transcribed spacer 2 of the nuclear rDNA cistron (ITS2), congruently recovered a robust phylogenetic structure. With ITS2 considerable sequence and secondary structure divergence existed among the eight species, but a combined sequence and secondary structure phylogenetic analysis confined to helix II of ITS2 corroborated relationships as inferred from the *rbc*L gene phylogeny. Intra-genomic divergence of ITS2 sequences was revealed in many strains. The ‘monophyletic species concept’, appropriate for microalgae without known sexual reproduction, revealed eight different species. Species boundaries established using the molecular-based monophyletic species concept were more conservative than the traditional morphological species concept. Within a species, almost identical chloroplast marker sequences (genotypes) were repeatedly recovered from strains of different origins. At least two species had widespread geographical distributions; however, within a given species, genotypes recovered from Antarctic strains were distinct from those in temperate habitats. Furthermore, the sequence diversity may correspond to adaptation to different types of habitats or climates.

**Conclusions:**

We established a method and a reference data base for the unambiguous identification of species of the common soil microalgal genus *Heterococcus* which uses DNA sequence variation in markers from plastid and nuclear genomes. The molecular data were more reliable and more conservative than morphological data.

## Background

**Figure 1:**
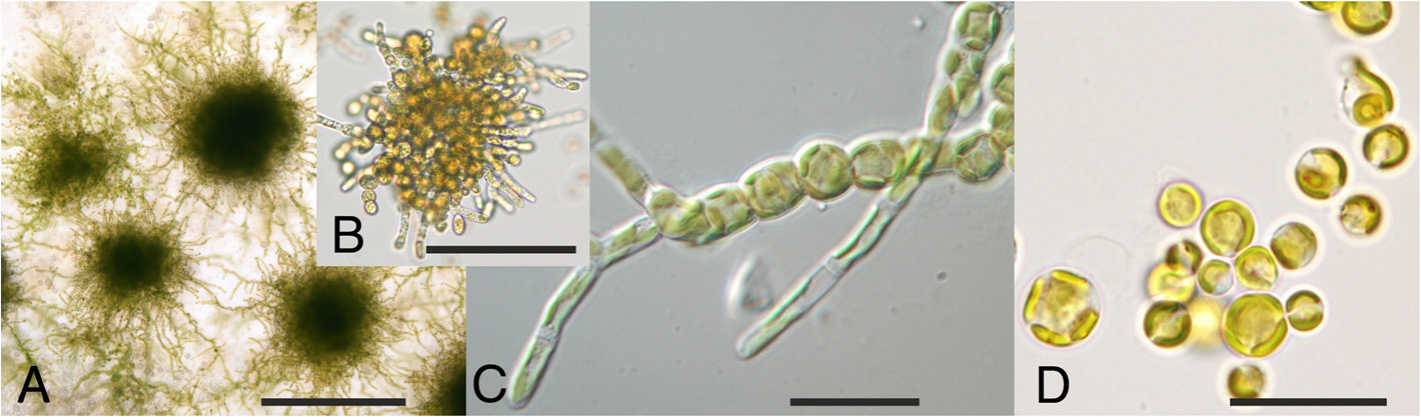
**Morphology of three strains of *Heterococcus viridis *in culture.** (**A**) Prostrate colonies produced by branched filaments on the surface of an agarized culture, 16 weeks old (strain B10). (**B**) Enlarged view of a young (4 weeks old) colony, liquid culture, strain SAG 835–7. (**C**) Enlarged filament, 6 week old agarized culture (strain MZ3-7). (**D**) Coccoid cells in a 4 weeks old liquid culture (strain SAG 835–7). Scale bar in (**A**) 500 μm, in (**B**) – (**D**) 20 μm.

*Heterococcus* is a genus of yellow-green microalgae (Xanthophyceae, Stramenopiles) that is common and widespread in soils of cold regions such as the Alps or Antarctica [1,2]. In addition to soils, three species have been reported from freshwater [3-6], and *Heterococcus* is the only xanthophyte known from lichen symbiosis [7,8]. *Heterococcus* produces extensively branched filaments when grown on agarized culture medium (Figure 1); however, in field samples it produces unicellular coccoid cells that are weakly connected. Perhaps uniquely for microalgal genera, all species have been described based upon isolates grown in culture and observed with a light microscope [1,2,6]. Without culturing, *Heterococcus* is often mistaken for other coccoid xanthophytes, eustigmatophytes or green algae. Sixty-one *Heterococcus* species have been described [9], and 51 species are recognized [10]. Extensive ultrastructural observations were undertaken by Lokhorst [2], but he reluctantly concluded that ultrastructural features were not sufficient to distinguish species. 

Sexual reproduction is unknown for *Heterococcus*, and therefore the biological species concept cannot be employed (e.g. [11]); only the morphological (typological) species concept has been used. That is, *Heterococcus* species identity is limited to light microscopic morphological characters interpreted within the extensive plasticity that is exhibited during culture studies [1,2,6]. For example, branching patterns are not present in very young or old cultures, and filament formation is suppressed (coccoid cells are produced) when cultures are grown at suboptimal temperature ranges [1] (Figure 1). Cladistic analysis of these morphological features would be extremely difficult because cell sizes, branching patterns, colony growth, chloroplast number and other features overlap extensively among the species, even when grown under optimum conditions.

**Figure 2:**
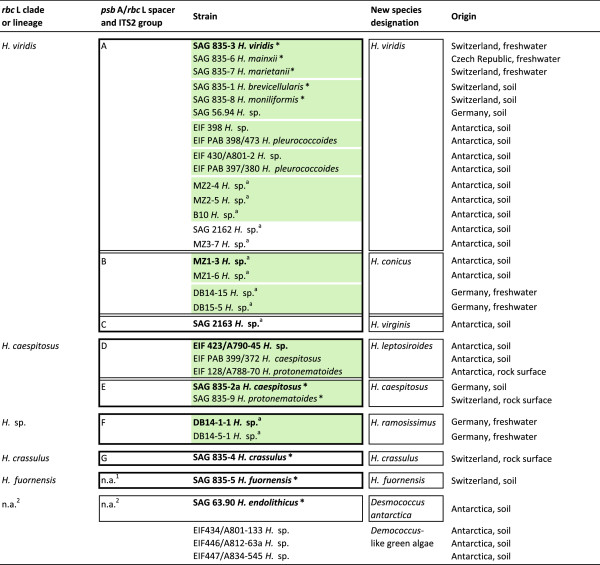
**The 33 strains identified as *Heterococcus *used in this study.** The *Heterococcus* strains are listed with their species names (where provided) from previous morphological analyses, their assignment to clades and lineages in the *rbc*L phylogeny (boxed with thick lines; see Figure 3), their assignments to a certain species recognized in this study (boxed with thin lines), their new species designations (see Discussion) and their geographic origin. Highlighted in green are genotypes, i.e. groups of strains exhibiting high sequence similarities (see text). Strains in bold letters represent cryopreserved epitypes (reference strains) designated for each species (see Discussion). An asterisk marks an authentic reference strain (see text). ^a^ marks those strains that have recently been isolated by us or were provided to us for this study; n.a.^1^, not applicable because the *psb*A/*rbc*L spacer sequence could not be determined (see text); n.a.^2^, not applicable because strains were identified as green algae (see text).

Molecular phylogenetic analysis is often a reliable alternative for identification of species; however, species diversity of *Heterococcus* using molecular markers was unstudied and no molecular reference data base existed. From only seven *Heterococcus* species DNA sequences had previously been reported, and all these sequences were from conserved molecular markers. The sequences revealed the probable monophyletic origin of the genus and its basal position within the Xanthophyceae, which was distinct from other filamentous members (e.g. *Tribonema, Vaucheria*) [12-15]. We used molecular phylogenetics, especially within the framework of the monophyletic species concept [16-18], to evaluate 33 culture strains identified as *Heterococcus* (Figure 2). Fourteen strains were originally identified to species level using morphology, and ten of those strains were authentic culture strains, i.e. the culture strains used to describe the species [1,3-5,19]. Unfortunately, the cultures used to describe all other species have been lost. For nine authentic strains, there are extended morphological descriptions with numerous illustrations produced by two independent authors [2-5]. We added 19 unidentified culture isolates, including twelve cultures recently isolated. Our goals were (1) to test boundaries of *Heterococcus* species as inferred from morphological features and (2) to establish a reference data base of strains unambiguously distinguished with DNA sequence data. We chose two highly variable molecular markers, i.e. the chloroplast-encoded *psb*A/*rbc*L spacer region [20,21] and the nuclear-encoded internal transcribed spacer 2 of the nuclear rDNA cistron [22-24], to examine species boundaries. We also determined full plastid-encoded *rbc*L gene sequences to infer the phylogenetic position of species. 

## Results

**Figure 3:**
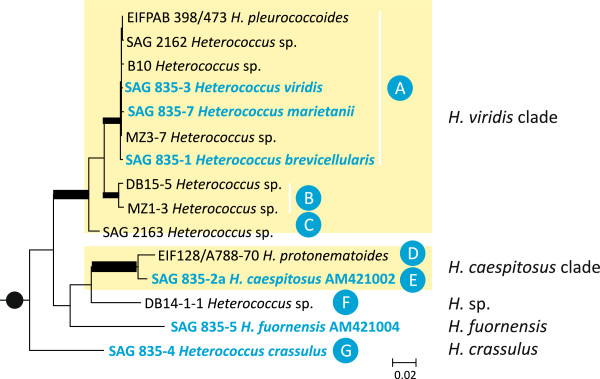
**Maximum likelihood (ML) phylogeny of *rbc*L gene sequences for 15*Heterococcus*strains.** Twelve other strains had sequences identical to one of the 15 shown (Additional file 3). Sequences without accession numbers are reported for the first time. Sequence names highlighted in blue indicate authentic reference strains; names used in the tree are those used to identify the original cultures (see text). Capital letters in filled blue circles indicate the seven species apart from *H. fuornensis* resolved by the *psb*A/*rbc*L spacer and ITS2 sequence analyses (see text). The names next to the tree represent clades and lineages recovered in the phylogenetic analyses. Thick lines indicate internal branches resolved by maximum likelihood, maximum parsimony, minimum evolution distance and Bayesian analyses and with significant statistical support (bootstrap >95%, posterior probability = 1.0). Black filled circle marks the branch indicating the monophyletic origin of *Heterococcus* that was significantly supported (bootstrap >95%, posterior probability = 1.0) except for the maximum likelihood analyses. The phylogeny shown is part of a larger ML phylogeny (calculated with GARLI v0.96 [25,26]) based on a *rbc*L data set (1325 bp long, 517/418 variable/parsimony informative sites) consisting of 15 *Heterococcus* sequences and 32 other Xanthophyceae sequences corresponding to clades C, B, T, and V as defined in [14] (see Additional file 1) as well as two outgroup taxa. Scale bar, substitutions per site.

Four of the strains, identified as *Heterococcus*, were green algae (Figure 2). These were not included in the rest of the study. The *rbc*L gene sequences were used to assess the phylogenetic relationships of the remaining 29 strains (Figure 3, Additional file 1). For 25 strains, PCR amplification was successful for the whole region from *psb*A (downstream), through the *rbc*L, through the *rbc*L/*rbc*S spacer and to the *rbc*S gene; therefore the full *rbc*L gene, 1467 base pairs long, was determined (Additional file 2). We failed to obtain full *rbc*L sequences for three authentic strains, *Heterococcus fuornensis* Vischer strain SAG 835–5, *H. caespitosus* Vischer strain SAG 835-2a, and *H. protonematoides* Vischer strain SAG 835–9, but we used available sequences (AM421004, AM421002 and AJ579575) for these three strains. Also, for strains DB14-15 and MZ1-6 the full *rbc*L failed to amplify. Fifteen different *rbc*L sequences were recovered among the 29 strains, which implies that the *rbc*L gene was identical among many strains (Additional file 3). Only the 15 different *rbc*L sequences were used for phylogenetic analyses (Figure 3, Additional file 1). Monophyly of *Heterococcus* was highly supported with all methods except maximum likelihood, and this confirmed the generic identity of the 29 strains. The analyses resolved two well supported clades, named “*H. caespitosus* clade” and “*H. viridis* clade”. In addition, there were three independent lineages representing *H. crassulus* Vischer*, H. fuornensis* and an unidentified strain (“*H.* sp.”). Relationships among the clades and lineages remained ambiguous (Figure 3, Additional file 1).

### *psb*A/*rbc*L spacer

To further examine the relationships, the *psb*A/*rbc*L spacer sequences were determined for 28 strains (*H. fuornensis* strain SAG 835–5 failed to amplify). The spacers varied greatly in length and primary sequences; the sequences could not be aligned across all strains. Nevertheless, two short sequence stretches were aligned across all strains. The first was 23 nucleotides at the 5’-end (pos. 78–99 of reference sequence *H. viridis* Chodat strain SAG 835–3, JX681220) and the second was 36 nucleotides at the 3’-end (pos. 312 – 347, same reference sequence).

In most *Heterococcus* strains the nucleotide length of the *psb*A/*rbc*L spacer ranged from 275 nucleotides (*H. caespitosus* strain SAG 835-2a,) to 289 nucleotides (*H.* sp. strain DB14-15). The sequence for *H. crassulus* strain SAG 835–4 was 1762 nucleotides, and the identical sequences for two strains, DB14-1-1 and DB14-5-1, were 2143 nucleotides. Sequence similarities further downstream grouped the strains into seven “spacer groups”, A – G, within which the *psb*A/*rbc*L spacers were identical or displayed only very few differences (Figure 2, Additional file 3). When mapped on the *rbc*L phylogeny, the strains of spacer groups A, B and C were included in the *H. viridis* clade, strains of spacer groups D and E fell in the *H. caespitosus* clade, and spacer groups F and G represented the lineages “*H.* sp.” and *H. crassulus* (Figure 3).

Between closely related groups or within a group, also other regions of the *psb*A/*rbc*L spacer sequences could be aligned. For example, strains of the *Heterococcus viridis* clade (groups A-C) had sequence regions that aligned well, but there were up to 28 nucleotide differences among them. In addition, there was a hypervariable region of different lengths (20–31 nucleotides, between pos. 172 and 193 of the reference sequence *H. viridis* SAG 835–3, JX681220) that was not alignable among the three groups, but clearly distinguished them from each other. In the *H. caespitosus* clade, i.e. between groups D and E, the *psb*A/*rbc*L spacers also aligned well over the entire lengths, but differed at 14 sequence positions and a single indel. Similarly, there was a maximum of 13 *psb*A/*rbc*L spacer sequence differences between strains of group A. In group A there were nine strains isolated from Antarctica (Figure 2). There were no more than two nucleotides difference among them when Antarctic strain MZ3-7 was not considered and the previously unidentified strain SAG 56.94, isolated from Germany, had just one to three sequence differences with the eight Antarctic isolates. Conversely, strain MZ3-7 was with seven to nine spacer differences more distant to the other eight Antarctic strains. Strain *H. brevicellularis* Vischer SAG 835–1 was the closest neighboring strain of strain MZ3-7; there were just 4 sequence positions different between both strains. Group B contained two Antarctic strains (MZ1-3, MZ1-6) that had identical spacers; Group B also contained two German strains (DB14-15, DB15-5) with identical spacers; however, the Antarctic strains differed at 4 positions when compared to the German strains. Finally, group D had three strains that had only one nucleotide difference, while two strains in group F had only two sequence differences.

### ITS2

Nuclear-encoded ITS2 sequences were determined for 28 strains as an independent assessment of the plastid-encoded sequences. *Heterococcus fuornensis* strain SAG 835–5 was successfully amplified and included; however, amplification failed for strain MZ1-6 and this strain was not included in the ITS2 analyses. Based upon alignment similarity, the ITS2 sequences formed the same groups that were recovered in the *psb*A/*rbc*L spacer analysis; therefore, we used the same group notation for both datasets. Within a spacer group, the ITS2 sequences and their secondary structures were easily aligned and rather similar; conversely, between spacer groups, the sequences and secondary structures were highly variable, i.e. they could be aligned with confidence only for a few short segments. The ITS2 sequences exhibited a considerable length variation of up to about 130 nucleotides between spacer groups. The shortest ITS2 had 285 nucleotides (strain SAG 2163 from group C; strain EIF 399/372 from group D); the longest sequence had 416 nucleotides (strains DB14-1-1, DB 14-5-1 from group F). Within each spacer group, the ITS2 sequences were relatively constant in length (variation < 10 nucleotides), except for group D where sequences were either short (285–287 nucleotides) or long (315–319 nucleotides), and the difference was due to an indel at the terminal end of helix III in the secondary structure model (see below; Additional file 4). The ITS2 sequence from *Heterococcus fuornensis*, which had a distinctive *rbc*L gene but could not be amplified for the *psb*A/*rbc*L spacer, showed little similarity to other spacer groups.

**Figure 4:**
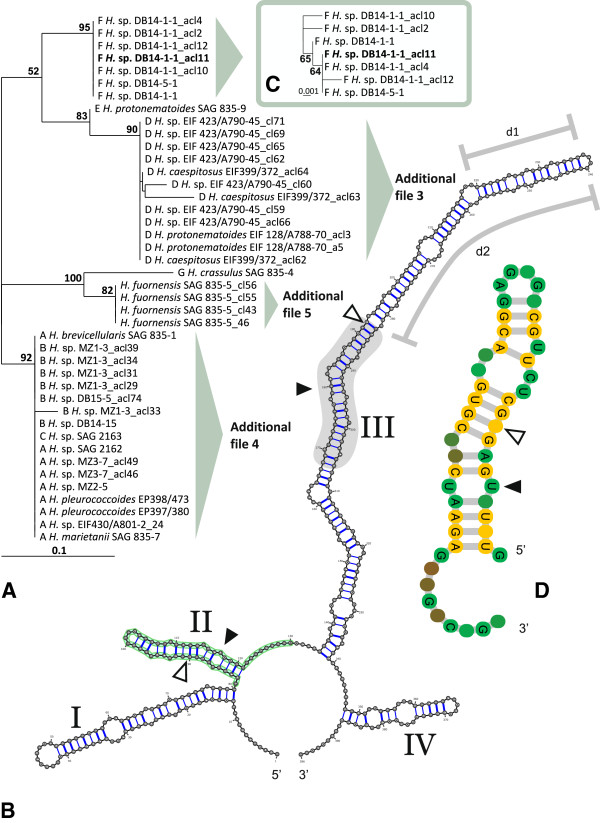
**ITS2 sequence and secondary structure phylogenetic analyses of 28 strains of *Heterococcus.*** (**A**) ProfDistS [27] sequence-structure NJ tree (unrooted) as derived from the multiple sequence-structure alignment of ITS2 helix II. Bootstrap values (100 pseudo-replicates) are mapped to the appropriate internodes. Branch lengths are drawn proportional to inferred changes. The template ITS2 variant used in B) is highlighted in bold. (**B**) ITS2 secondary structure of ITS2 variant DB14-1-1_acl11 (group F, *H. ramosissimus*) used for homology modeling of helix II (shaded) for all strains of *Heterococcus*. The secondary structure was visualized with VARNA [28]. Helices are numbered I–IV. Typical ITS2 motifs are highlighted by filled arrowheads. Open arrowheads mark positions of two CBCs that distinguish groups D (= *H. leptosiroides*) and E (= *H. caespitosus*). An additional conserved region throughout all strains of *Heterococcus* is indicated by a cloud (see text). In contrast to the template structure the region d1 is deleted in four strains (group D, see Additional file 4). The region d2 is deleted in all other strains not classified in group F. (**C**) Subtree as obtained by using the complete sequence-structure information from helices I-IV (template highlighted in bold). Further subtrees as derived using clade specific structural templates (helices I-IV) are provided as Additional files 4, 5, 6. (**D**) Visualization of the complete sequence-structure alignment used to generate the tree as shown in A). Consensus structure (51%) of helix II for all ITS2-sequences obtained from the complete multiple sequence-structure alignment without gaps. Sequence conservation is indicated from red (not conserved) to green (conserved). Nucleotides which are 100% conserved in all sequences are written as A, U, G or C. Nucleotide bonds which are 100% conserved throughout the alignment are marked in yellow. Note the U-U mismatch. The figure was generated with 4SALE [29].

The inferred RNA secondary structures folded into the common core structure known for eukaryotes [23] which consisted of four helices with the third being the longest and helix IV the shortest (Figure 4, Additional files 4, 5, 6). Because of the high sequence length variation there was not a single ITS2 secondary structure from which the secondary structure models of the remaining sequences could be deduced using homology modeling. Only helix II could be modeled throughout the set of sequences independent of the used sequence-structure pair. However, within each group complete secondary structures could be obtained by homology modeling (Figure 4, Additional files 4, 5, 6). Throughout the set of sequences, conserved regions were restricted to the entire helix II (pos. 86–125 of reference sequence *H. viridis* SAG 835–3, JX681147), which had a constant length of 40 nucleotides, and a segment of about 50 nucleotides (pos. 165–189 and 205–228 of the same reference sequence) located at or close to the distal end of helix III (Figure 4, Additional files 4, 5, 6). It was followed by an extended terminal end of the helix III of 45 and 133 nucleotides in spacer groups D and F, whereas the corresponding sequence region in other spacer groups comprised of six (*H. fuornensis* strain SAG 835–5, no assigned group) to 18 nucleotides (spacer group E). That means there was a continuous lengthening/shortening of the ITS2 helix III within *Heterococcus* (Figure 4, Additional files 4, 5, 6). Another conserved ITS2 region useful to distinguish groups among *Heterococcus* strains was an unpaired sequence segment (~12 nucleotides) adjacent to helix II (Figure 4; pos. 126–137 of reference sequence *H. viridis* SAG 835–3, JX681147). It separated *H. crassulus* SAG 835–4, *H. fuornensis* SAG 835–5, and two clusters of strains from each other. The one cluster comprised the strains from groups A-C, the other the strains from groups D-F. Within each cluster the sequence segments were invariant. 

Multiple copies of ITS2 were recovered in eight strains (from groups A, B, D, F and G; Additional file 2), i.e. there were no clear sequence reads possible without cloning. Four to 12 clones per strain were sequenced and this revealed up to seven ITS2 variants per strain (Additional file 2). Differences between ITS2 variants consisted of one to seven sequence positions and a few small indels (< 5 nucleotides); they were mostly located in helices I, IV and the basal part of helix III preceding the conserved segment. In groups B and D differences between ITS2 variants were also located in the conserved helix II. In group D three out of the ten detected ITS2 variants were lacking the extended 45 nucleotides long terminal end of helix III. These shorter variants were present in all three strains of group D or in about half (10) out of the sequenced 21 clones, while the longer ITS2 variants were retrieved only from two strains, EIF 423/A790-45 and EIF 128/A788-70.

The ITS2 phylogenetic analyses were confined to helix II (with adjacent unpaired conserved region, pos. 85–134 of reference sequence *H. viridis* SAG 835–3, JX681147) for assessing relationships among all studied strains. The sequence alignment was with 50 positions relatively short; it contained no more than 14/9 variable/parsimony informative sites and just nine sequences were not identical with others. However, a well-resolved phylogeny was obtained when secondary structure was considered in addition to primary structure information (Figure 4). The resolved helix II sequence groups were congruent with the groups recovered in the *rbc*L phylogeny (see spacer group letters on Figure 4). A common origin of *H. crassulus* with *H. fuornensis* was well supported in the unrooted ITS2 (helix II) phylogeny, and this contrasted with the *rbc*L phylogeny where the relationships of both were unresolved (Figure 3). Also an unrooted (maximum likelihood) phylogeny of only *Heterococcus rbc*L gene sequences did not support the common origin of both species (not shown). The helix II phylogenetic tree resolved a close relationship of groups D and E (as the *rbc*L phylogeny, *H. caespitosus* clade in Figure 3), but at the same time both groups were clearly separated species because there were two CBCs [23,24] in helices II and III (Figure 4, Additional file 4); also their helices I and IV could not be aligned. No resolution was provided within the *H. viridis* clade, i.e. among spacer groups A, B and C (Figure 4). The complete ITS2 sequence was used to produce phylogenetic trees for individual spacer groups or *rbc*L clades. For example, then within the *rbc*L *H. viridis* clade the spacer groups A-C were resolved (Additional file 5). Within group A, both variants of strain MZ3-7 shared a common origin and were separated from other strains of the group. The three authentic strains, *Heterococcus viridis* SAG 835–3, *H. mainxii* SAG 835–6, and *H. marietanii* SAG 835–7, shared identical ITS2 sequences with each other (Additional file 7). Similarly, the ITS2 sequences of the two Antarctic strains EIF 398 and EIF PAB 398/473 were identical (Figure 2, Additional file 3). Two authentic strains, *H. brevicellularis* SAG 835–1 and *H. moniliformis* SAG 835–8, and one unidentified strain (SAG 56.94) shared identical ITS2 sequences except for a short indel (4 nucleotides) in helix IV. Another congruence with the chloroplast-encoded data was within group E where the ITS2 sequences of two authentic strains, *H. caespitosus* SAG 835-2a and *H. protonematoides* SAG 835–9, were identical. Conversely, within group B no differentiation among strains was possible due to the extensive radiation of multiple ITS2 variants of strain MZ1-3 (Additional file 5). Similarly, group D had extensive radiation of ITS2 variants and no relationships among strains were resolved (Additional file 4). Here the shorter variants of both strains EIF 423/A790-45 and EIF PAB 399/372 were intermixed among each other; they formed two independent lineages distinct from a clade comprising the variants with extended terminal end of helix III. Within group F no clear distinction of the two strains DB14-1-1 (with multiple variants) and DB14-5-1 was provided (Figure 4).

## Discussion

### Monophyletic species concept

Our results show that morphological features do not characterize species; for example, we found that five authentic culture strains – used in the original descriptions for the five species – had nearly identical DNA sequences and ITS2 secondary structures. Furthermore, we found other examples where authentic strains or identified strains were synonymous with another species (see below). Almost all *Heterococcus* species have been described using the same morphological approach, we have examined all existing authentic culture strains, and we find that morphological species descriptions are inadequate for this asexual genus. We conclude that morphological features characterize only individuals, not species. Therefore, we must apply a different species concept for *Heterococcus*.

The ‘monophyletic species concept’ of Johansen and Casamatta [18], which is derived from the ‘phylogenetic (autapomorphic) species concept’ of Mishler and Theriot [16,17], is easily applied to asexual species when molecular data are available. In our study, the DNA sequences and ITS2 secondary structure comparisons recovered a clear and robust phylogenetic structure for the 29 *Heterococcus* strains. Eight groups of sequences were repeatedly recovered using three different molecular markers; sequences within each group were very similar or identical while those between groups were highly variable. Using the monophyletic species concept, we recognize these groups as eight distinct species, and we identify previously unidentified strains and environmental clones to species level.

In a previous study, the *rbc*L gene and *psb*A/*rbc*L spacer were used, in conjunction with the monophyletic species concept, to define species in the Tribonemataceae, another asexual lineage of filamentous Xanthophyceae [21]. In that study, strains of the same species formed a monophyletic clade in the maximum likelihood *rbc*L gene phylogeny, and strains within the same species differed by less than 10 nucleotides. Within each species, the *psb*A/*rbc*L spacer was easily aligned, and within species variation was limited to single nucleotide differences and short indels. As with our study, the entire spacer could not be aligned between species. Therefore, the molecular-based monophyletic species concept identifies species in the same way for both studies.

The original iconotypes used to nomenclaturally anchor all *Heterococcus* names consist of ink drawings of various morphological features. We have shown that these morphological features are not reliable for species identity, and ink drawings are very limited for reference. In some cases, neotype material was dried and deposited in a herbarium [2], but this too is ambiguous because in at least one case, the wrong culture was used (see below) and because the material does not clearly separate species (still based upon morphology). Therefore, the names are herein further anchored with epitypes to avoid all ambiguity. The epitypes here designated are cryopreserved culture strains that can be re-investigated. The nomenclatural details are summarized below.

### Taxonomy and nomenclature

Group A strains differed by no more than five sequence positions (one nonsynonymous substitution) in their *rbc*L genes, and the *psb*A/*rbc*L spacer regions aligned well over their entire lengths, with no more than 11 sequence differences. Their ITS2 sequences also aligned well over their entire lengths and there were no more than eight ITS2 sequence positions different. Therefore, we regard group A as a single species, *Heterococcus viridis*, which is the type species for the genus. It is noteworthy that we used Chodat’s [19] authentic strain, SAG 835–3 [3,30]. Group A also contained four additional species that were based upon authentic strains, *H. brevicellularis, H. mainxii, H. marietanii* and *H. moniliformis*[4,5]. We conclude that interpretations of largely overlapping morphological features, which were used to establish these as separate species, are not taxonomically sound; therefore, we consider these to be heterotypic synonyms of *H. viridis* (Figure 2, Additional files 3 and 7). Previously, Lokhorst [2] found that three of these strains were morphologically almost indistinguishable and he considered them as varieties. Group A also includes two strains previously identified as *H. pleurococcoides* Pitschmann [1]. However, the two strains were not authentic strains, and we cannot completely conclude that *H. pleurococcoides* is a heterotypic synonym of *H. viridis.* In addition, eight unidentified strains are now identified as *H. viridis* based on our study (Figure 2).

***Heterococcus viridis*Chodat in*Bull. Herb. Boissier***, **ser. 2, 8: p. 81 (1907).**

Neotype: Material (authentic culture strain SAG 835-3) deposited in Nationaal Herbarium Nederland, Leiden University (L) by G.M. Lokhorst in *Taxonomic Studies in the Genus* Heterococcus*. Cryptogamie Studies* Vol. 3, p. 40. (1992).

Epitype designated here: Cryopreserved culture strain SAG 835-3, deposited in the Sammlung von Algenkulturen (SAG), Universität Göttingen, Germany.

Heterotypic synonyms:

*Heterococcus brevicellularis* Vischer in *Ergeb. Wiss. Unters. Schweiz. Nationalparkes, N.F*. 1: p. 504; pl. 4, figures 1–3; figure 17A, d-f; figure 18. (1945).

*Heterococcus mainxii* Vischer in *Ber. Schweiz. Bot. Ges*. 47: p. 233; figures 4–6 (1937).

*Heterococcus marietanii* Vischer in *Ber. Schweiz. Bot. Ges*. 47: p. 235; figure 7 (1937).

*Heterococcus moniliformis* Vischer in *Ber. Schweiz. Bot. Ges*. 47: p. 238; figures 8–9 (1937).

*Heterococcus marietanii* Vischer var. *moniliformis* Lokhorst in *Taxonomic Studies in the Genus* Heterococcus*. Cryptogamie Studies* Vol. 3, p. 39. (1992).

Two authentic strains in group E, *H. caespitosus* SAG 835-2a and *H. protonematoides* SAG 835–9, were identical when considering the three markers. We recognize group E as a single species. *H. caespitosus* was described first [3], and therefore has nomenclatorial priority over *H. protonematoides*[5], which becomes a heterotypic synonym.

***Heterococcus caespitosus*Vischer in*Ber. Schweiz. Bot. Ges.*45: p. 391, figures 4–10 (1936).**

Iconotype: Figures 4-10 in Vischer, W. *Ber. Schweiz. Bot. Ges.* 45: 372-410 (1936).

Neotype: Material (authentic culture strain SAG 835-9) deposited in Nationaal Herbarium Nederland, Leiden University (L) by G.M. Lokhorst in *Taxonomic Studies in the Genus* Heterococcus*. Cryptogamie Studies* Vol. 3, p. 12. (1992). Note: The culture strain used to designate the neotype material belonged to *Heterococcus protonematoides*, not *H. caespitosus*; see Lokhorst (1992, p. 12).

Epitype designated here: Cryopreserved culture strain SAG 835-2a, deposited in the Sammlung von Algenkulturen (SAG), Universität Göttingen, Germany.

Heterotypic synonyms:

*Heterococcus protonematoides* Vischer in *Ergeb. Wiss. Unters. Schweiz. Nationalparkes, N.F*. 1: p. 502, pl. 2, figures 1–3; figures 15,16. (1945).

For group G, *H. crassulus* was represented by an authentic strain, and we accept this as a recognized species. Similarly, for an unnamed group (see Figure 2), *H. fuornensis* was represented by an authentic strain, and therefore we recognize this as a distinct species.

***Heterococcus crassulus*Vischer in*Ergeb. Wiss. Unters. Schweiz. Nationalparkes, N.F*. 1: p. 503, pl. 3, figures 1–3; figures 17, 17A, l-o (1945).**

Iconotype: Figure 17 in Vischer, W. *Ergeb. Wiss. Unters. Schweiz. Nationalparkes, N.F*. 1: 479-512 (1945).

Neotype: Material (authentic culture strain SAG 835-4) deposited in Nationaal Herbarium Nederland, Leiden University (L) by G.M. Lokhorst in *Taxonomic Studies in the Genus* Heterococcus*. Cryptogamie Studies* Vol. 3, p. 12. (1992).

Epitype designated here: Cryopreserved culture strain SAG 835-4, deposited in the Sammlung von Algenkulturen (SAG), Universität Göttingen, Germany.

***Heterococcus fuornensis*Vischer in*Ergeb. Wiss. Unters. Schweiz. Nationalparkes, N.F*. 1: p. 506, pl. 5, figures 1–3; figure 17A, a-c; figure 19 (1945).**

Iconotype: Figure 19 in Vischer, W. *Ergeb. Wiss. Unters. Schweiz. Nationalparkes, N.F*. 1: 479-512 (1945).

Neotype: Material (authentic culture strain SAG 835-5) deposited in Nationaal Herbarium Nederland, Leiden University (L) by G.M. Lokhorst in *Taxonomic Studies in the Genus* Heterococcus*. Cryptogamie Studies* Vol. 3, p. 12. (1992).

Epitype designated here: Cryopreserved culture strain SAG 835-5, deposited in the Sammlung von Algenkulturen (SAG), Universität Göttingen, Germany.

In group B, there were no more than nine different sequence positions (one nonsynonymous substitution) among the complete *rbc*L sequences and only four nucleotide differences among the *psb*A/*rbc*L spacers. Strains of group B formed a well-supported monophyletic clade independent of other groups/species in the *rbc*L phylogeny (Figure 3, Additional file 1) as well as phylogenetic analysis of the whole ITS2 region (Additional file 5). Therefore, we recognize group B as a distinct species. Placing a scientific name on group B (species B) is problematic because our study included all existing authentic cultures. Our molecular data, which were rigorously analyzed with phylogenetic methods, contradict species distinctions based upon non-rigorous intuition using highly variable morphological features, and we conclude that our rigorous analyses are more scientifically sound. Nonetheless, there are 61 named species, and perhaps group (species) B belongs to one of those species. If we simply propose a new name, then we are defying the intent of the International Code of Botanical Nomenclature (or any other Code). Therefore, we simply apply four of the oldest names used in [6] for group (species) B and the three other groups (C, D, F) which contained no authentic strains. We assume that none of these names is in contradiction with the morphology of the strains we designate to represent the four species. We argue that establishing axenic cultures and examining filaments at a certain age of a culture time (as it has been done to define species of *Heterococcus* previously [2-6]) is a poor way to identify species and this does not allow field samples to be identified to species. With *Heterococcus* growth in culture is a measure of meaningless differences and there is no hope that morphology will ever be useful when trying to put a name on these four groups (species). We suggest that close phylogenetic relationship with defined reference (epitype) strains as well as genetic distance from corresponding strains of other species, evidenced by *rbc*L gene phylogenies and differences in the *psb*A/*rbc*L spacers are appropriate to identify the species. Secondary structure of ITS2 constitutes an additional autapomorphic feature to define species of *Heterococcus*. We use *Heterococcus conicus* Pitschmann as name for group (species) B.

***Heterococcus conicus*Pitschmann*in*Pitschmann, H.*Nov. Hed.*5 (3/4), p. 498, plate 96, Figures 11-16, (1963)**

Iconotypes: Plate 96, Figures 11-16, in Pitschmann, H. *Nov. Hed.* 5 (3/4), (1963)

Neotype: Material (culture V 111) deposited in Nationaal Herbarium Nederland, Leiden University (L) by G.M. Lokhorst in *Taxonomic Studies in the Genus* Heterococcus*. Cryptogamie Studies* Vol. 3, p. 12. (1992).

Epitype designated here: Cryopreserved culture strain MZ1-3, deposited in the Sammlung von Algenkulturen (SAG), Universität Göttingen, Germany.

Group C consisted of a single strain, SAG 2163 (Figure 2), which formed a distinct lineage in the *rbc*L and full ITS2 phylogenies (Figure 3, Additional files 1 and 4). It was also distinct in its *psb*A/*rbc*L spacer from *H. viridis* and *H. conicus* which were the closest relatives with SAG 2163. Therefore, we recognize group C as a distinct species and we use *Heterococcus virginis* Pitschmann as name. Two unidentified lichen photobionts share identical partial *rbc*L sequences (JN573801 and JN573802; [8]) and these differed by only one nucleotide from SAG 2163. Therefore, we assign these lichen photobionts to *H. virginis* as well.

***Heterococcus virginis*Pitschmann*in*Pitschmann, H.*Nov. Hed.*5 (3/4), p. 497, plate 96, Figures 1-5, (1963)**

Iconotypes: Plate 96, Figures 1-5, in Pitschmann, H. *Nov. Hed.* 5 (3/4), (1963).

Epitype designated here: Cryopreserved culture strain SAG 2163 deposited in the Sammlung von Algenkulturen (SAG), Universität Göttingen, Germany.

Group D comprised three strains with no nucleotide difference in the *rbc*L and a single in the *psb*A/*rbc*L spacer. In the ITS2 phylogeny, the three strains could not be distinguished due to different ITS2 variants that are intermixed (Additional file 4). Strains of group D exhibit a unique ITS2 secondary structure with a rather long helix III with considerable length variation at its terminal end (Additional file 4). Despite being closely related to *H. caespitosus* (group E) in the *rbc*L phylogeny (Figure 3 and Additional file 1) there are two CBCs in ITS2 that separate group D strains from the latter species. Consequently, we recognize group D as a distinct species, *Heterococcus leptosiroides* Pitschmann. One environmental clone sequence from Antarctic soils (AJ580925) shared full sequence identity in *rbc*L gene with group D strains, and therefore, we conclude that the environmental clone belongs to *H. leptosiroides*. Group D included strains identified as *H. caespitosus* and *H. protonematoides* based on morphology; however, neither was an authentic culture and again we consider identification based on

***Heterococcus leptosiroides*Pitschmann*in*Pitschmann, H.*Nov. Hed.*5 (3/4), p. 497, plate 96, Figures 6–10, (1963).**

Iconotypes: Plate 96, Figures 6-10, in Pitschmann, H. *Nov. Hed.* 5 (3/4), (1963).

Epitype designated here: Cryopreserved culture strain EIF 423/A790-45 deposited in the Sammlung von Algenkulturen (SAG), Universität Göttingen, Germany.

Finally, group F contained two strains with fully identical complete *rbc*L sequences and two differences in their *psb*A/*rbc*L spacers. In the ITS2 phylogeny the two strains could not be distinguished due to the variation of multiple copies (Figure 4C). The group F strains had a unique ITS2 secondary structure with a particularly long helix III (Figure 4B). Group F forms an independent lineage within the *Heterococcus* clade in the *rbc*L phylogeny (Figure 3, Additional file 1). Consequently, we recognize group F as a distinct species and use *Heterococcus ramosissimus* Pitschmann as name.

***Heterococcus ramosissimus*Pitschmann*in*Pitschmann, H.*Nov. Hed.*5 (3/4), p. 499, plate 97, Figures 1–4, (1963)**

Iconotypes: Plate 97, Figures 1-4, in Pitschmann, H. *Nov. Hed.* 5 (3/4), (1963).

Epitype designated here: Cryopreserved culture strain DB14-1-1 deposited in the Sammlung von Algenkulturen (SAG), Universität Göttingen, Germany.

The authentic strain of *H. endolithicus* was described by Darling and coworkers [1], 195/A790-35 (accessioned as strain SAG 63.90 by the SAG culture collection), but our study revealed that it represents a green alga, i.e. a close relative of *Desmococcus* species (Trebouxiophyceae) (Figure 2). Our microscopic investigation of SAG 63.90 revealed the same morphology as described previously [1]. Significantly, this morphology is somewhat similar to the morphology of *Desmococcus*[10], and this makes us confident that SAG 63.90 still represents the original isolate. Despite Darling and coworkers [1] having reported a “typical xanthophycean plastid structure” based on electron microscopy, they already considered *H. endolithicus* distinct from all other *Heterococcus* species because it did not form long filaments. In addition, three more strains from Antarctic soils were also identified as *Desmococcus*-like green algae (Figure 2). Therefore, we exclude *H. endolithicus* from the genus *Heterococcus* and propose a new nomenclatural combination for this authentic strain, but unfortunately we cannot apply the specific epithet (*endolithicus*) because the name *Desmococcus endolithicus* Broady & Ingerfeld already exists [31]. Therefore, we propose an avowed substitute name:

***Desmococcus antarctica*(Darling & Friedmann) Rybalka, Wolf, Andersen & Friedl*comb. nov.***

Basionym: *Heterococcus endolithicus* Darling & Friedmann In Darling et al. *J Phycol***23:** 599, Figures 2a-c, 3. (1987).

Epitype designated here: Cryopreserved culture strain SAG 63.90 deposited in the Sammlung von Algenkulturen (SAG), Universität Göttingen, Germany.

### Infraspecific Variation and Geographical Distribution

Our relatively small sample of 29 *Heterococcus* strains already showed eight groups (= eight species). Within the five species for which multiple strains were available, the *psb*A/*rbc*L spacer sequences even resolved groups of strains with nearly identical sequences (genotypes; Figure 2). Strains with identical, or nearly identical, sequences were repeatedly found in our relatively small sample of *Heterococcus* strains and, importantly, established at different times from geographically distant localities. This implies that the number of species within *Heterococcus* might be rather limited. The same genotypes were confined to certain habitats (soil or freshwater) and geographical regions (Europe or Antarctica). For example, *H. viridis* strains SAG 835–3, SAG 835–6 and SAG835-7 were collected from freshwater habitats in Europe while all other strains of the species were from soil in Europe or Antarctica (Figure 2); they represent a distinct subgroup (genotype) within the species. Similarly, two strains of *H. conicus* were collected from freshwater in Europe (DB14-15, DB15-5) whereas the other two *H. conicus* strains were collected from Antarctic soil (MZ1-3, MZ1-6; Figure 2). We draw two conclusions. First, the two species are geographically widespread and will grow where suitable habitats exist. Second, genotypes of those growing in freshwater are distinct from those growing in soil. The sample size is exceedingly small, but there is a suggestion that our molecular data are separating populations within both species that have distinctly different habitats.

We also note that half of the *Heterococcus* genotypes in our sample originated from Antarctica but not a single genotype was shared between Antarctic and European strains, i.e. none of the Antarctic *Heterococcus* strains shared identical *psb*A/*rbc*L spacer sequences with the European strains. A previous study showed that Antarctic strains within a single species of the xanthophyte *Xanthonema* were distinguished from their temperate counterparts by only few nucleotides for the highly variable *psb*A/*rbc*L spacers [21]. Therefore, our findings for *Heterococcus* support the view that the Antarctic and temperate strains of xanthophyte species represent different populations of a single species. That is, the Antarctic strains of a given species share their own common evolutionary histories, implying that there was only one (relatively recent) colonization event in Antarctica for each species. Alternatively, if multiple colonization events occurred, then the invasions were too recent to produce significant divergence [32,33].

### ITS2 sequence features

Our ITS2 sequences are, to our knowledge, the first ITS2 sequences available for Xanthophyceae. Given that available ITS2 sequence information for stramenopile algae is still limited, two aspects of the *Heterococcus* ITS2 sequences appear unusual, but might be useful for taxonomy. First, in *Heterococcus* ITS2 lengths were approximately 300 nucleotides long in most strains; group F (*H. ramosissimus*) sequences were almost 400 bps. The average length of ITS2 across all eukaryotes is about 210 bps as inferred from the ITS2 database IV [34]. In group D, two size classes occurred, i.e. either ~250 or ~300 bps, due to a large indel at the terminal end of helix III. Other stramenopile algal groups, the Bacillariophyceae and Phaeophyceae, show a bimodal distribution of their ITS2 sequence lengths, i.e. around 250/290 bps and around 250/350 bps, respectively. Second, the ITS2 sequences were rather variable, i.e. only few and rather short sequence segments were alignable with confidence across the eight *Heterococcus* species. Such a high sequence variation among species of a single genus is unusual, at least as compared to genera and species of green algae where ITS2 has been revealed as a reliable molecular marker already many times, e.g. [35-38]. Finally, because the ITS2 rDNA sequences were so variable in *Heterococcus*, it is not possible to safely define compensatory base changes (CBCs), which can be deduced only from well aligned sequences. CBCs in conserved regions of the helices of ITS2 have been proposed for distinguishing microalgal species when sexual reproduction is unknown [23,24]. However, the concept of CBCs does not imply that two strains lacking CBCs must belong to the same species. That is, there may be other criteria that define microalgal species.

The high ITS2 sequence variability is in line with our maximum likelihood (GARLI and RAxML) analyses that had weak support for the monophyletic origin of the genus (Additional file 1). The monophyletic origin of *Heterococcus* was also weakly supported by a multiple gene phylogenetic analyses of photosynthetic stramenopiles that included three of our *Heterococcus* species [15]. Therefore, our results may suggest that more data (and better taxon sampling) are required to firmly demonstrate the monophyly of *Heterococcus*, or they may suggest that some of the species defined in our study belong to a separate, and sister, genus.

## Conclusions

Application of the monophyletic species concept using the highly variable chloroplast-encoded *psb*A/*rbc*L spacer, the more conserved plastid *rbc*L gene, and the nuclear-encoded ITS2 provided a reference data base for unambiguous identification of the common cold soil microalga *Heterococcus*. Eight species were recognized and characterized at the molecular level. Previous taxonomic studies relied entirely on morphological features produced in cultures; our data will facilitate diversity assessments that are independent of culturing. In addition, the PCR amplification approach for the *psb*A/*rbc*L spacer is specific for Xanthophyceae. Using the new reference data base, partial sequences of the *psb*A/*rbc*L spacer and/or ITS2 may already be sufficient for the assignment of a new strain to a certain species. There are some difficulties; amplification of the *psb*A/*rbc*L spacer may be hampered by length variations, and sequence analyses of ITS2 may be complicated by multiple variants per strain. Using the monophyletic species concept, our species are mostly in contrast to those defined by the morphological (typological) species concept. We conclude that the extensive morphological plasticity displayed in culture cannot be interpreted without rigorous methods (e.g. cladistics), and the largely overlapping morphological characteristics make cladistic analysis very difficult or impossible. The identical, but highly variable, sequences that were repeatedly recovered among the species, suggest that the species diversity of *Heterococcus* is not extensive, especially considering the repetition that occurred in our small sampling from Europe and Antarctica. The observed sequence changes within a species may reflect adaptations to different types of habitats or climates and distinguish geographically widely separated strains.

## Methods

### Culture strains

Twenty three culture strains were received from the SAG culture collection [39,40]; five strains were provided by other workers in the field. Another five isolates (strains MZ1-3, MZ1-6, MZ2-4, MZ2-5, MZ3-7) were newly established using methods described previously [21] from Antarctic soil samples, i.e. the forefield of Baranowski Glacier, King George Island (collected December 12 2008 by M. Olech). Strains MZ1-3 and MZ1-6 were from the same sample, about 5 m from the glacier (62°12′34,9˝S, 58°26^′^55,7˝W) at 10 m a.s.l. Strains MZ2-4 and MZ2-5 also were from a single sample, a frontal moraine (62°12^′^34.4˝S- 58°26^′^50.2˝W) at 16 m a.s.l. Strain MZ3-7 was from a basal moraine (62°12^′^33.4˝S – 58°26^′^41.1˝W) at 6 m a.s.l. Antarctic strain B10 (provided by A. Massalski) was isolated also from King George Island, but from transect B near Ecology Glacier about 370 m farther inland; [41]). Four isolates (DB14-1-1, DB14-5-1, DB14-15 and DB15-5; provided by K. M. Mohr) were from cyanobacteria-dominated biofilms covering rocks at two neighboring locations of the main spring of the tufa-forming karst-water creek, Deinschwanger Bach, located at the western margin of the Franconian Alb, approximately 30 km ESE of Nürnberg, Germany (49°23’N, 11°28’E) [42]. The ten new isolates have been accessioned by the SAG culture collection under strain numbers as given in Additional file 2.

### DNA extraction, PCR amplification and sequencing of strains

DNA was isolated from fresh cultures as in [21]. For determining sequences of the plastid-encoded *psb*A/*rbc*L spacer which lies upstream of the *rbc*L gene, i.e. between the *psb*A and *rbc*L genes, and full-length sequences of the *rbc*L gene the PCR approach of Andersen and Bailey [20] modified to amplify the target sequence in one piece [21] was used. The 5’ primer *psb*A5 [20] or Xan2F [21], anchored in the *psb*A gene, and the 3’ primer RS3 [20] placed in *rbc*S (downstream of *rbc*L) were used. However, for strains with extremely long *psb*A/*rbc*L spacers, PCR amplification was in two overlapping fragments, i.e. with primer pairs *psb*A5 and X5RG (the reverse complement of primer X5FG [21]) and Xan3F [21] and RS3 [20]. For amplification of ITS2, PCR primers Xits2F (5’ –GCTACACTCTGACACCTG -3’; which binds at the 5’-end of the 18S rRNA gene, i.e. pos. 1462–1477 of reference sequence AM490822 *H. viridis* SAG 835–3, and LR1850 [43] were used to amplify a rDNA fragment that expanded from 3’-end of SSU downstream to the 5‘-end of the LSU rDNA. The same cycling parameters were used for all PCR reactions as described previously [21]. PCR products were purified using Invisorb Spin PCRapid Kit (Invitek, Berlin, Germany) or MSB Spin PCRapace Kit (Invitek, Berlin, Germany). Sequence determination of the *psb*A/*rbc*L spacer was as previously [21], but complemented by nine additional primers to obtain the sequences of the extremely long *psb*A/*rbc*L spacers present in some *Heterococcus* strains, i.e. hetnew_F (5’–GGTACAACTGAYCAATT-3’), het_F (5’–GGTGGTACAATTGGYCATCCAGA-3’), spacer2R (5’–ATTCGAGTACGCTCTTGTA-3’), DB_F (5’–GGCAAGCCTTTCACTCTTGAT–3’), DB_R (5’-CCACCCGGATTTAAAAGAGTT-3’), DB_F2 (5’–TTCGATACGGGAAACAACTT–3’), DB_R2 (5’–GATCCTTTGGTTCAACTTAGAAGA–3’), SAG_F (5’–CAAGCTTCGACTGAGGCTT–3’), and SAG_R (5’–ATTGCAAGGCAAGCCTTG–3’). The latter two sequencing primers were used only for *H*. *crassulus* strain SAG 835–4, the “DB” primers only with the two isolates DB14-1-1 and DB14-5-1. The *rbc*L sequences were checked against the NCBI gene sequence database using nucleotide BLAST (blastn) [44,45] to confirm that they were Xanthophyceae. For four strains from the SAG culture collections no PCR products of plastid-encoded markers as described above could be obtained and then a portion of the nuclear-encoded 18S rRNA gene was sequenced with primer 895R [46] after PCR amplification with primers preferentially binding to green algal rDNA, primers 20 F [8] and CH1750R [46] and checked against the NCBI gene sequence database. For ITS2 sequence determination, the sequencing primers were 5.8SbF and 5.8SbR [47], 1800 F [43] and ITS4Xan (5’-TCCTCCGCTTAGTTATATGC-3’), which was a modification of primer ITS4 [48]. In several cases no clear sequence reads were obtained, even after repeated PCR and sequencing attempts, due to multiple copies of the ITS2 which varied in primary sequences (see Results). Then cloning of the PCR products was performed with the TOPO TA cloning kit and the pCR2.1-TOPO vector (Invitrogen, Carlsbad, CA, USA). Ligations were transformed into competent *E. coli* TOP 10 cells as supplied by the manufacturer. In the plasmid screening, white *E. coli* colonies containing correct DNA insertions were identified by direct amplification of the inserted DNA fragment with a vector-specific primer set M13F/M13R. The ITS fragments were re-amplified from M13F/M13R PCR products with primer pair Xits2F/LR1850 as described above or the clones were cultivated overnight in LidBac reaction tubes (Qiagen, Hilden, Germany) with 1 ml LB medium containing 100 μg ampicillin and plasmid DNA was prepared from the clones with a NucleoSpin-Plasmid kit (Macherey and Nagel, Düren, Germany) following manufacturer’s instructions. Sequencing reactions were performed with the Dye Terminator Cycle Sequencing v3.1 kit (Applied Biosystems, Darmstadt, Germany) and separated on an ABI Prism 3100 (Applied Biosystems, Darmstadt, Germany) sequencer. The sequences were assembled using the program SeqAssem [49]. For GenBank accession numbers of newly determined sequences for the 29 *Heterococcus* strains see Additional file 2; the accession numbers for the four green algal sequences determined in this study are JX681197 - JX681200.

### Chloroplast-encoded marker analysis

The chloroplast-encoded marker sequences (from 3’-end of *psb*A downstream to 5’-end of *rbc*S) were manually aligned using Bioedit [50] and Seaview [51] editors from which the *rbc*L sequence alignment used for the phylogenetic analyses was extracted. The *rbc*L sequence alignment was constructed using 15 of the sequences newly determined for *Heterococcus* in this study to which 32 other sequences available for the Xanthophyceae clades C, B, T, and V as defined previously [14] were added (Additional file 1). The two phaeophycean sequences *Fucus vesiculosus* NC016735 and *Ectocarpus* sp. AY372978 were employed to root the phylogeny. The alignment was subjected to distance, maximum-parsimony (MP) and maximum-likelihood (ML) approaches. ModelTest 3.7 [52] used in conjunction with PAUP* 4b10 [53] determined that the GTR+I+G model [54] provided the best fit to the data according to the AIC criterion with estimations of nucleotide frequencies (A = 0.2859, C = 0.1447, G = 0.1981, T = 0.3714), a rate matrix with six different substitution types, assuming a heterogeneous rate of substitutions with a gamma distribution of variable sites, number of rate categories = 4, shape parameter α = 0.8249 and proportion of invariable sites (*pinvar*) of 0.4977. This model was used for the minimum evolution distance (ME) approach performed with PAUP* 4b10 (DNA distances set to maximum likelihood) and the maximum likelihood ML (approach) using GARLI v0.96 [25,26]. A complementary ML phylogeny construction was done with the program RAxML [55], using the GTR+Γ+I model and with 100 bootstrap replicates. Neighbor-joining distance (NJ) phylogenies were constructed in connection with the “HKY85 model” [56] with PAUP* 4b10. For ME and maximum parsimony (MP) tree reconstruction (PAUP* 4b10) a heuristic search procedure with 10 random input orders of sequences and TBR were employed to find the best tree. Best scoring trees were held at each step. In MP analyses, the sites were weighted (RI over an interval of 1–1000). Bootstrap resampling was performed on NJ, ME, MP with 1000 replications and 2000 replications on ML GARLI trees. For the Bayesian analysis the program MrBayes version 3.1.2 [57] was used with procedures as described earlier [58].

### Nuclear-encoded ITS2 sequence-structure analysis

Using hidden Markov models (HMMs) nuclear ITS2 sequences have been annotated according to [59]. One ITS2 sequence from each group A, D, F and *H. fuornensis* strain SAG 835–5 was used for secondary structure prediction. Based on minimum free energy ITS2 secondary structures were directly folded with the help of the “RNAstructure” software [60,61] and manually corrected. The four sequence-structure pairs were used as templates for homology modeling of the remaining 39 secondary structures [62]. In accordance to [63] the phylogenetic analysis followed the procedure outlined in [23,34,64,65]: automatically, a multiple sequence-structure alignment was generated in 4SALE v1.7 [29,66], i.e. either partial (Figure 4) or full (Additional files 4, 5, 6) sequences and their secondary structures were synchronously aligned, making use of an ITS2 sequence-structure specific scoring matrix [66,67]. Based simultaneously on the primary sequence and the secondary structure information, phylogenetic relationships were reconstructed using NJ through in conjunction with an ITS2 sequence-structure specific general time reversible (GTR) substitution model as implemented in ProfDistS v0.9.9 [27,67]. Bootstrap support [68] was estimated based on 100 pseudo-replicates (Figure 4, Additional files 4, 5, 6). Trees were visualized using Treeview [69].

## Abbreviations

SAG: Culture Collection of Algae at Göttingen University, Germany (*Sammlung von Algenkulturen der Universität Göttingen, Göttingen*, Germany); ML: Maximum likelihood; MP: Maximum parsimony; ME: Minimum evolution; NJ: Neighbor joining.

## Competing interests

The authors declare that they have no competing interests.

## Authors’ contribution

NR conceived and designed the study, carried out the molecular genetic studies, established cultures, participated in the phylogenetic analyses including ITS2 secondary structure models and drafted the manuscript. MW performed the analyses of ITS2 secondary structure models and ITS2 phylogenetic analyses, contributed to interpretation of the results and was involved in critically revising the manuscript. RAA developed all aspects regarding taxonomy of *Heterococcus*, participated in the interpretation of the molecular data and critically revised the manuscript. TF and RAA wrote the final manuscript. TF participated in all phylogenetic analyses and interpretation of the molecular data. All authors have contributed on the manuscript drafting, read and approved the final manuscript.

## Supplementary Material

Additional file 1 **Maximum likelihood (ML) phylogeny of *rbc*L gene sequences for *Heterococcus*and other members of Xanthophyceae.** The phylogeny was calculated with the programme GARLI v0.96 [25,26] based on a *rbc*L data set (1325 bp long, 517/418 variable/parsimony informative sites) consisting of 15 *Heterococcus* and 32 other Xanthophyceae sequences (corresponding to clades C, B, T, and V as defined in [14]) as well as two sequences from Phaeophyceae as outgroup. Scale bar, substitution per site. Numbers mapped to internodes are bootstrap values from 2000 replicates, only values >70% have been recorded. The phylogeny in this Figure includes the phylogeny of 15 *Heterococcus* strains shown in Figure 3 (highlighted). The inserted table lists bootstrap values mapped to internodes of the *Heterococcus* clade using six different analysis methods (see text). Scale bar, substitution per site. Click here for file

Additional file 2 **DNA sequences newly determined for 29 *Heterococcus *strains and their GenBank sequence accession numbers.** For the *psb*A/*rbc*L spacer and full *rbc*L gene all determined sequences are listed, for ITS2 only those sequences that were different from each other. (p), only *psb*A/*rbc*L spacer and partial full *rbc*L gene could be determined; (a), already made available previously; n.a., not applicable. Click here for file

Additional file 3 **Groups of *Heterococcus *strains with fully identical *rbc*L and/or *psb*A/*rbc*L spacer sequences.** Strains marked in bold were used for the *rbc*L phylogeny (Figure 3, Additional file 1). Species assignment is according to the new species designation as in Figure 2 (see Discussion). Click here for file

Additional file 4 **ITS2 sequence and secondary structure phylogenetic analyses of three strains of *Heterococcus *group D* (H. leptosiroides).*** (**A**) ProfDistS [27] sequence-structure NJ tree (unrooted) as derived from the multiple sequence-structure alignment of ITS2 helices I-IV recovered for strains of group D, *H. leptosiroides*. Bootstrap values based on 100 pseudo-replicates are mapped to the appropriate internodes. Branch lengths are drawn proportional to inferred changes. The template ITS2 variant used in B) is highlighted in bold. Scale bar, substitutions per site. (**B**) ITS2 secondary structure of ITS2 variant EIF 423/A790-5_cl65 used for homology modeling of secondary structures for all strains of group D (*H. leptosiroides*). The secondary structure was visualized with VARNA [28]. Helices are numbered I–IV. Four strains indicated by an asterisk are devoid of the apical part of helix III. An arrowhead indicates the highly conserved GGU motif 5’ to the apex of helix III. A cloud highlights the segment of helix III conserved across all studied strains. Open arrowheads mark positions of two CBCs that distinguish groups D and E. Click here for file

Additional file 5 **ITS2 sequence and secondary structure phylogenetic analyses of twelve strains of *Heterococcus *groups A-C *(H. viridis, H. conicus, H. virginis).*** (**A**) ProfDistS [27] sequence-structure NJ tree (unrooted) as derived from the multiple sequence-structure alignment of ITS2 helices I-IV recovered for strains of the *H. viridis* clade, i.e. groups A-C, *H. viridis, H. conicus* and *H. virginis*. Bootstrap values based on 100 pseudo-replicates are mapped to the appropriate internodes. Branch lengths are drawn proportional to inferred changes. The template ITS2 variant used in B) is highlighted in bold. Scale bar, substitutions per site. (**B**) Secondary structure of ITS2 variant *H. viridis* EIF 430/A801-2_24 used for homology modeling of secondary structures for all strains of *Heterococcus* groups A-C. The secondary structure was visualized with VARNA [28]. Helices are numbered I–IV. An arrowhead indicates the highly conserved GGU motif 5’ to the apex of helix III. A cloud highlights the segment of helix III conserved across all studied strains. Click here for file

Additional file 6 **ITS2 sequence and secondary structure phylogenetic analyses of *Heterococcus fuornensis *strain SAG 835–5.** (**A**) ProfDistS [27] sequence-structure NJ tree (unrooted) of ITS2 variants recovered from strain *H. fuornensis* SAG 835–5 as derived from the multiple sequence-structure alignment of ITS2 helices I-IV. Bootstrap values based on 100 pseudo-replicates are mapped to the appropriate internodes. Branch lengths are drawn proportional to inferred changes. The template ITS2 variant used in B) is highlighted in bold. Scale bar, substitutions per site. (**B**) Secondary structure of ITS2 variant SAG 835-5_46 used for homology modeling of secondary structures for all ITS2 variants of the same strain. The secondary structure was visualized with VARNA [28]. Helices are numbered I–IV. An arrowhead indicates the highly conserved GGU motif 5’ to the apex of helix III. A cloud highlights the segment of helix III conserved across all studied strains. Click here for file

Additional file 7 **DNA sequence differences among five authentic strains of *Heterococcus *group A.** Distance matrices with number of sequence position differences from the *rbc*L gene, the *psb*A/*rbc*L spacer and ITS2 between the five authentic strains of *Heterococcus* group A (assigned to *H. viridis* , see text). In brackets, the total number of differences found with a certain molecular marker among the five strains. An asterisk marks the strain that is distinct from others by the presence of a “GCAA” indel in helix IV of ITS2. (DOCX 15 kb) Click here for file
