# Supplementary figures and images for: Congruence of chloroplast- and nuclear-encoded DNA sequence variations used to assess species boundaries in the soil microalga Heterococcus (Stramenopiles, Xanthophyceae)
Source: BMC Evol Biol. 2013 Feb 13;13:39. doi: 10.1186/1471-2148-13-39 (PMC3598724; doi:10.1186/1471-2148-13-39)

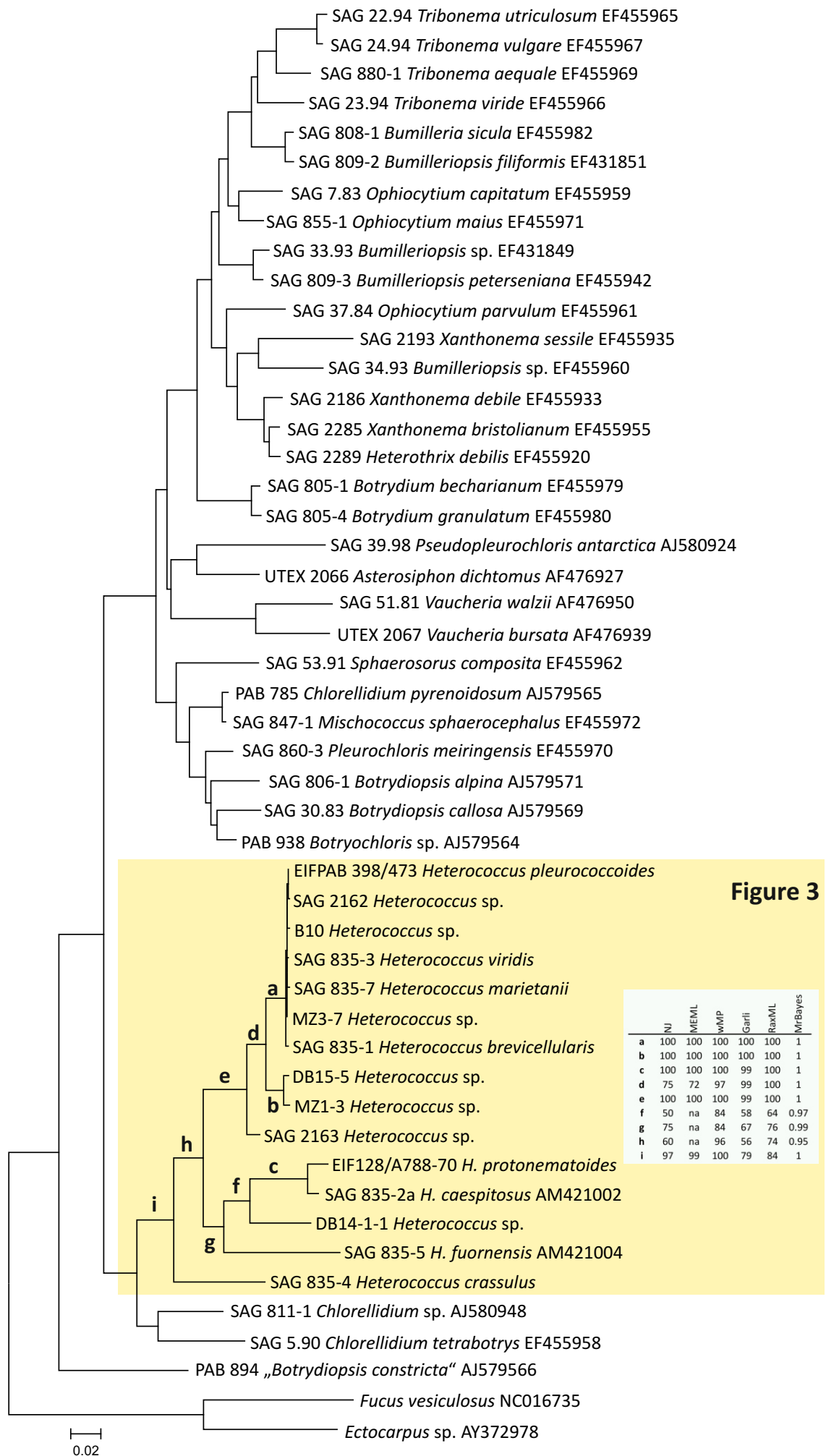

**Figure 3**

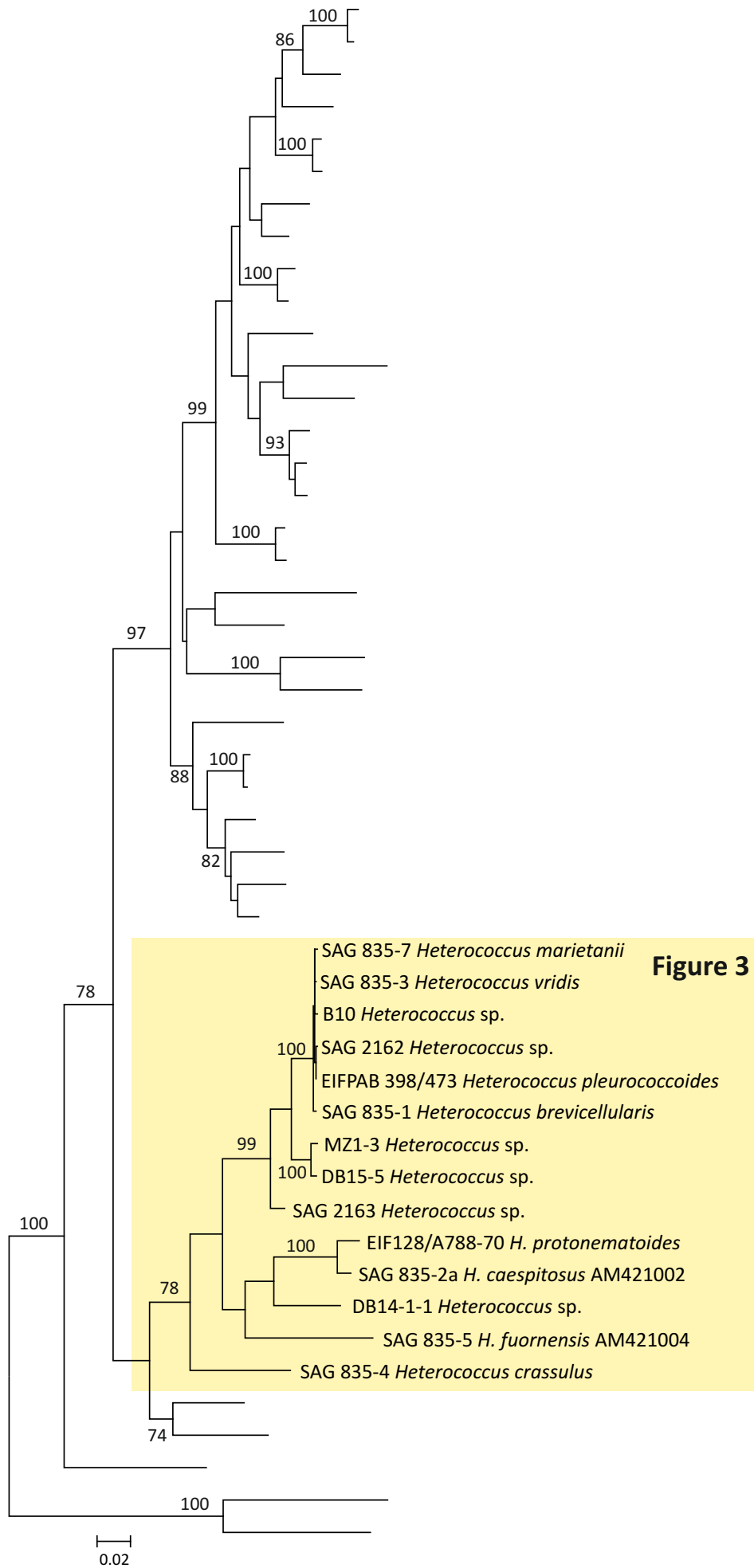

Supplement: Additional file 1 — Maximum likelihood (ML) phylogeny of rbcL gene sequences for Heterococcusand other members of Xanthophyceae. The phylogeny was calculated with the programme GARLI v0.96 [25,26] based on a rbcL data set (1325 bp long, 517/418 variable/parsimony informative sites) consisting of 15 Heterococcus and 32 other Xanthophyceae sequences (corresponding to clades C, B, T, and V as defined in [14]) as well as two sequences from Phaeophyceae as outgroup. Scale bar, substitution per site. Numbers mapped to internodes are bootstrap values from 2000 replicates, only values >70% have been recorded. The phylogeny in this Figure includes the phylogeny of 15 Heterococcus strains shown in Figure 3 (highlighted). The inserted table lists bootstrap values mapped to internodes of the Heterococcus clade using six different analysis methods (see text). Scale bar, substitution per site. [file 1471-2148-13-39-S1.pdf]

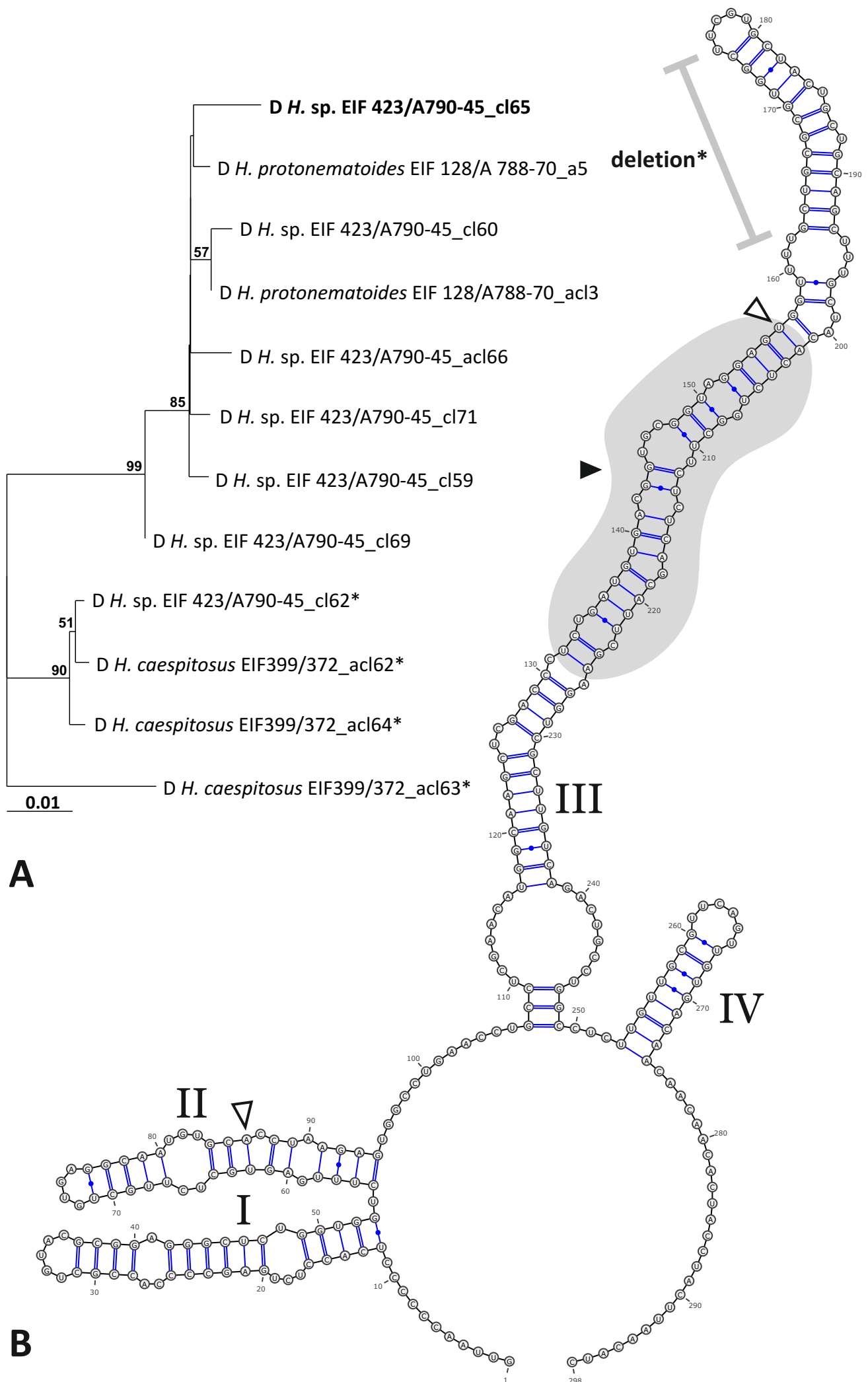

Supplement: Additional file 4 — ITS2 sequence and secondary structure phylogenetic analyses of three strains of Heterococcus group D (H. leptosiroides). (A) ProfDistS [27] sequence-structure NJ tree (unrooted) as derived from the multiple sequence-structure alignment of ITS2 helices I-IV recovered for strains of group D, H. leptosiroides. Bootstrap values based on 100 pseudo-replicates are mapped to the appropriate internodes. Branch lengths are drawn proportional to inferred changes. The template ITS2 variant used in B) is highlighted in bold. Scale bar, substitutions per site. (B) ITS2 secondary structure of ITS2 variant EIF 423/A790-5_cl65 used for homology modeling of secondary structures for all strains of group D (H. leptosiroides). The secondary structure was visualized with VARNA [28]. Helices are numbered I–IV. Four strains indicated by an asterisk are devoid of the apical part of helix III. An arrowhead indicates the highly conserved GGU motif 5’ to the apex of helix III. A cloud highlights the segment of helix III conserved across all studied strains. Open arrowheads mark positions of two CBCs that distinguish groups D and E. [file 1471-2148-13-39-S4.pdf]

**A**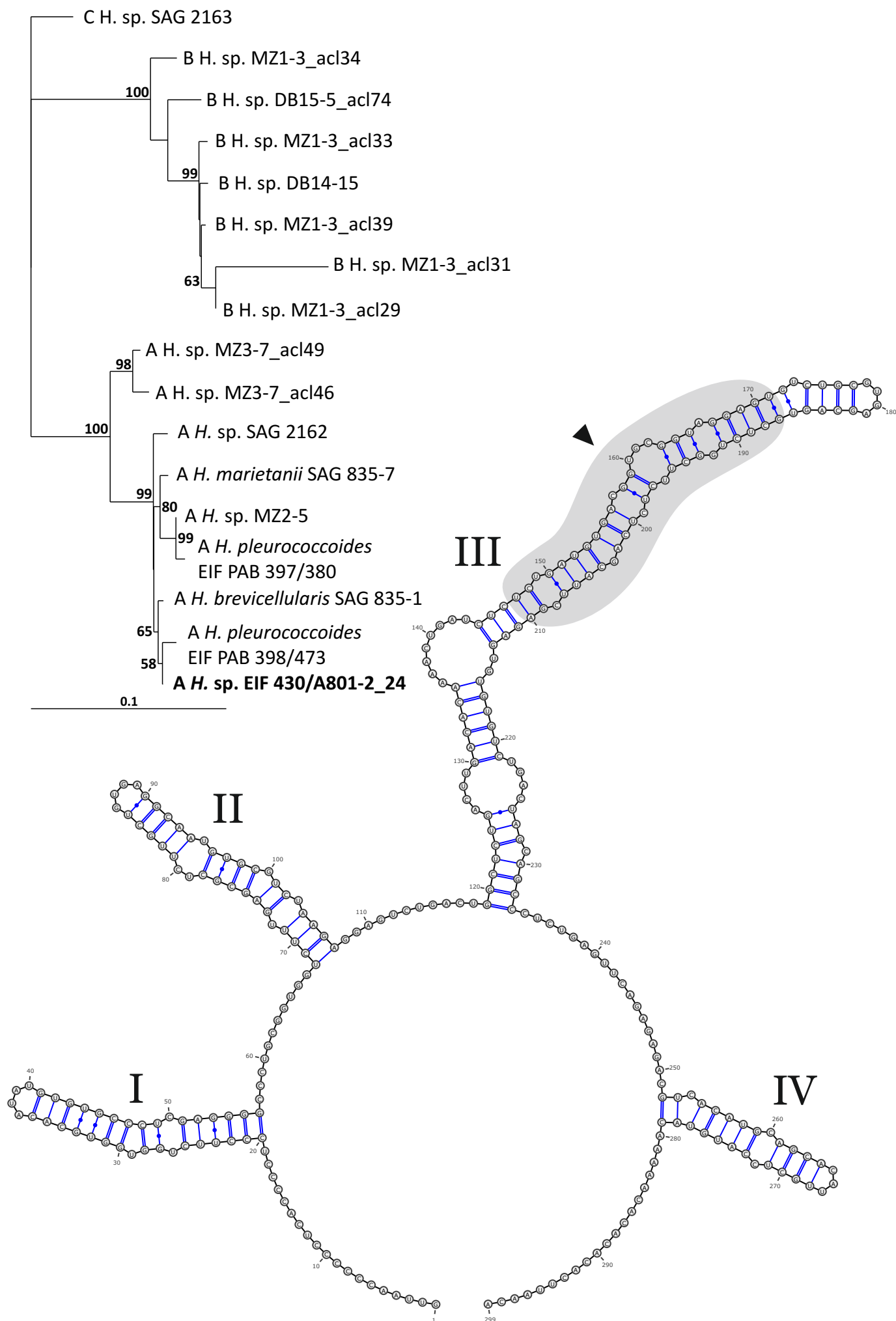**B**

Supplement: Additional file 5 — ITS2 sequence and secondary structure phylogenetic analyses of twelve strains of Heterococcus groups A-C (H. viridis, H. conicus, H. virginis). (A) ProfDistS [27] sequence-structure NJ tree (unrooted) as derived from the multiple sequence-structure alignment of ITS2 helices I-IV recovered for strains of the H. viridis clade, i.e. groups A-C, H. viridis, H. conicus and H. virginis. Bootstrap values based on 100 pseudo-replicates are mapped to the appropriate internodes. Branch lengths are drawn proportional to inferred changes. The template ITS2 variant used in B) is highlighted in bold. Scale bar, substitutions per site. (B) Secondary structure of ITS2 variant H. viridis EIF 430/A801-2_24 used for homology modeling of secondary structures for all strains of Heterococcus groups A-C. The secondary structure was visualized with VARNA [28]. Helices are numbered I–IV. An arrowhead indicates the highly conserved GGU motif 5’ to the apex of helix III. A cloud highlights the segment of helix III conserved across all studied strains. [file 1471-2148-13-39-S5.pdf]

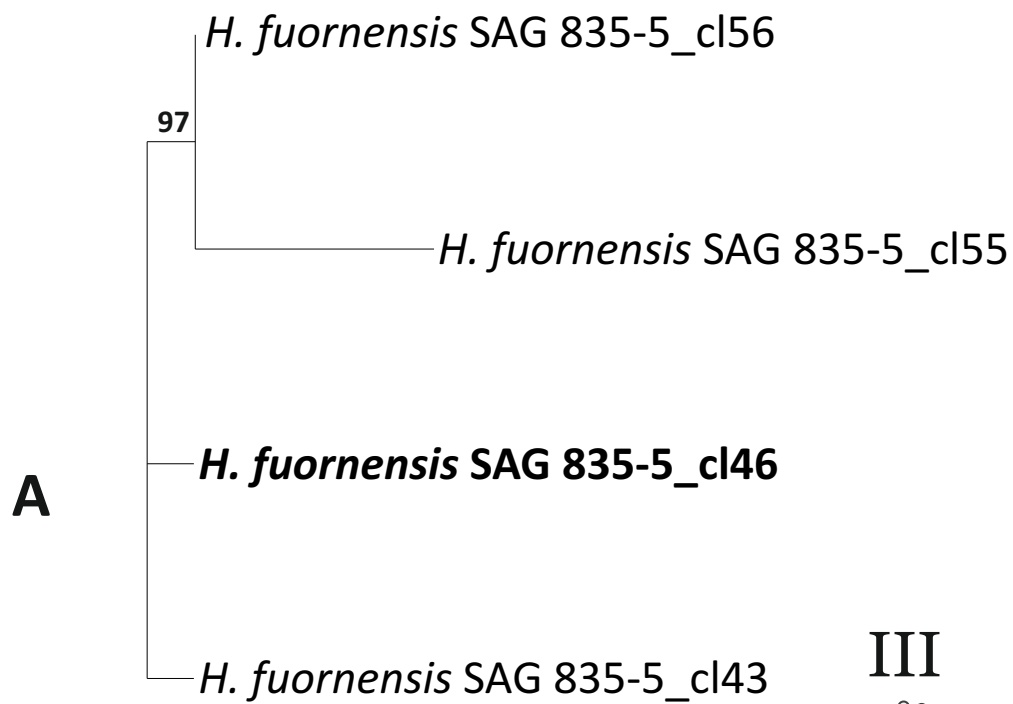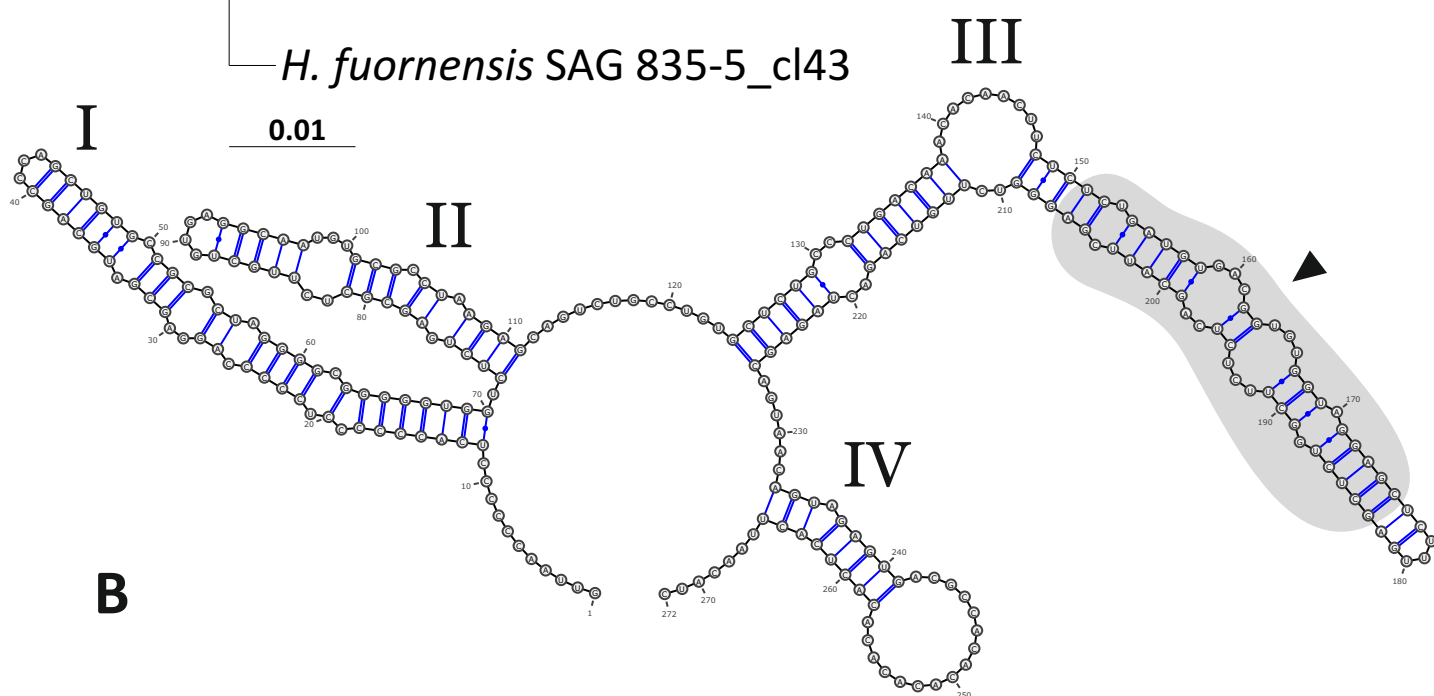

Supplement: Additional file 6 — ITS2 sequence and secondary structure phylogenetic analyses of Heterococcus fuornensis strain SAG 835–5. (A) ProfDistS [27] sequence-structure NJ tree (unrooted) of ITS2 variants recovered from strain H. fuornensis SAG 835–5 as derived from the multiple sequence-structure alignment of ITS2 helices I-IV. Bootstrap values based on 100 pseudo-replicates are mapped to the appropriate internodes. Branch lengths are drawn proportional to inferred changes. The template ITS2 variant used in B) is highlighted in bold. Scale bar, substitutions per site. (B) Secondary structure of ITS2 variant SAG 835-5_46 used for homology modeling of secondary structures for all ITS2 variants of the same strain. The secondary structure was visualized with VARNA [28]. Helices are numbered I–IV. An arrowhead indicates the highly conserved GGU motif 5’ to the apex of helix III. A cloud highlights the segment of helix III conserved across all studied strains. [file 1471-2148-13-39-S6.pdf]
