# Supplementary material for: Congruence of chloroplast- and nuclear-encoded DNA sequence variations used to assess species boundaries in the soil microalga Heterococcus (Stramenopiles, Xanthophyceae)
Source: BMC Evol Biol. 2013 Feb 13;13:39. doi: 10.1186/1471-2148-13-39 (PMC3598724; doi:10.1186/1471-2148-13-39)
Supplement: Additional file 2 — DNA sequences newly determined for 29 Heterococcus strains and their GenBank sequence accession numbers. For the psbA/rbcL spacer and full rbcL gene all determined sequences are listed, for ITS2 only those sequences that were different from each other. (p), only psbA/rbcL spacer and partial full rbcL gene could be determined; (a), already made available previously; n.a., not applicable. [file 1471-2148-13-39-S2.docx]

| **Strain** | **psbA/rbcLspacer and rbcL** | **ITS2 variant** | **ITS2** |
| --- | --- | --- | --- |
| SAG 835-3 | JX681220 |  | JX681147 |
| SAG 835-6 | JX681222 |  | JX681148 |
| SAG 835-7 | JX681223 |  | JX681149 |
| SAG 835-1 | JX681218 |  | JX681150 |
| SAG 835-8 | JX681224 |  | JX681151 |
| SAG 56.94 | JX681226 |  | JX681152 |
| EIF 398 | JX681207 |  | JX681153 |
| EIF 430/A801-2 | JX681209 |  | JX681154 |
| EIF PAB 398/473 | JX681211 |  | JX681155 |
| EIF PAB 397/380 | JX681210 |  | JX681156 |
| MZ2-4 (= SAG MZ2-4) | JX681215 |  | JX681157 |
| MZ2-5 (= SAG MZ2-5) | JX681216 |  | JX681158 |
| B10 (= SAG B10) | JX681201 |  | JX681159 |
| SAG 2162 | EF426795 (a) |  | JX681160 |
| MZ3-7 (= SAG MZ3-7) | JX681217 | MZ3-7_acl46 | JX681161 |
|  |  | MZ3-7_acl49 | JX681162 |
| MZ1-6 | JX681214 (p) |  | n.a. |
| MZ1-3 (= SAG 3334) | JX681213 | MZ1-3_acl29 | JX681163 |
|  |  | MZ1-3_acl31 | JX681164 |
|  |  | MZ1-3_acl33 | JX681165 |
|  |  | MZ1-3_acl34 | JX681166 |
|  |  | MZ1-3_acl39 | JX681167 |
| DB14-15 (= SAG 3335) | JX681204 (p) |  | JX681168 |
| DB15-5 (= SAG 3336) | JX681205 | DB15-5_acl74 | JX681169 |
| SAG 2163 | EF455945 (a) |  | JX681170 |
| EIF PAB 399/372 | JX681212 | EIF PAB 399/372_acl62 | JX681171 |
|  |  | EIF PAB 399/372_acl63 | JX681172 |
|  |  | EIF PAB 399/372_acl64 | JX681173 |
| EIF 423/A790-45 | JX681208 | EIF 423/A790-45_cl59 | JX681174 |
|  |  | EIF 423/A790-45_cl60 | JX681175 |
|  |  | EIF 423/A790_45_cl62 | JX681176 |
|  |  | EIF 423/A790-45_cl65 | JX681177 |
|  |  | EIF 423/A790-45_cl66 | JX681178 |
|  |  | EIF 423/A790-45_cl69 | JX681179 |
|  |  | EIF 423/A790-45_cl71 | JX681180 |
| EIF 128/A788-70 | JX681206 | EIF 128/A788-70_a5 | JX681181 |
|  |  | EIF 128/A788-70_acl3 | JX681182 |
| SAG 835-2a | JX681219 (p) |  | JX681183 |
| SAG 835-9 | JX681225 (p) |  | JX681184 |
| DB14-1-1 (= SAG 3337) | JX681202 | DB14-1-1 | JX681185 |
|  |  | DB14-1-1_acl2 | JX681186 |
|  |  | DB14-1-1_acl4 | JX681187 |
|  |  | DB14-1-1_acl10 | JX681188 |
|  |  | DB14-1-1_acl11 | JX681189 |
|  |  | DB14-1-1_acl12 | JX681190 |
| DB14-5-1 (= SAG 3338) | JX681203 |  | JX681191 |
| SAG 835-4 | JX681221 |  | JX681192 |
| SAG 835-5 | n.a. | SAG 835-5_cl43 | JX681193 |
|  |  | SAG 835-5_cl43 | JX681194 |
|  |  | SAG 835-5_cl55 | JX681195 |
|  |  | SAG 835-5_cl56 | JX681196 |
