# Supplementary material for: Congruence of chloroplast- and nuclear-encoded DNA sequence variations used to assess species boundaries in the soil microalga Heterococcus (Stramenopiles, Xanthophyceae)
Source: BMC Evol Biol. 2013 Feb 13;13:39. doi: 10.1186/1471-2148-13-39 (PMC3598724; doi:10.1186/1471-2148-13-39)
Supplement: Additional file 3 — Groups of Heterococcus strains with fully identical rbcL and/or psbA/rbcL spacer sequences. Strains marked in bold were used for the rbcL phylogeny (Figure 3, Additional file 1). Species assignment is according to the new species designation as in Figure 2 (see Discussion). [file 1471-2148-13-39-S3.docx]

|  |  |  |
| --- | --- | --- |
|  | rbcL identical | psbA/rbcL spacer identical |
| *H. viridis* | **SAG 835-7 *H. marietanii*** | SAG 835-7 *H. marietanii* |
|  | SAG 835-6 *H. mainxii* | SAG 835-6 *H. mainxii* |
|  |  |  |
|  | **SAG 835-1 *H. brevicellularis*** |  |
|  | SAG 835-8 *H. moniliformis* |  |
|  | SAG 56.94 |  |
|  |  | EIF 398 |
|  | EIF 398 | EIF PAB 398/473 |
|  | **EIF PAB 398/473** |  |
|  | EIF PAB 397/380 | EIF PAB 397/380 |
|  | EIF 430/A801-2 | EIF 430/A801-2 |
|  |  |  |
|  | MZ2-4 | MZ2-4 |
|  | MZ2-5 | MZ2-5 |
|  | **MZ3-7** | B10 |
|  |  |  |
| species B | **MZ1-3** |  |
|  | DB14-15 |  |
|  |  |  |
| species D | **EIF 128/A788-70** | EIF 128/A788-70 |
|  | EIF 423/A790-45 | EIF 423/A790-45 |
|  | EIF PAB 399/372 |  |
|  |  |  |
| *H. caespitosus* | **SAG 835-2a *H. caespitosus*** | SAG 835-2a *H. caespitosus* |
|  | SAG 835-9 *H. protonematoides* | SAG 835-9 *H. protonematoides* |
|  |  |  |
| species F | **DB 14-1-1** |  |
|  | DB 14-5-1 |  |
