# Supplementary material for: Congruence of chloroplast- and nuclear-encoded DNA sequence variations used to assess species boundaries in the soil microalga Heterococcus (Stramenopiles, Xanthophyceae)
Source: BMC Evol Biol. 2013 Feb 13;13:39. doi: 10.1186/1471-2148-13-39 (PMC3598724; doi:10.1186/1471-2148-13-39)
Supplement: Additional file 7 — DNA sequence differences among five authentic strains of Heterococcus group A. Distance matrices with number of sequence position differences from the rbcL gene, the psbA/rbcL spacer and ITS2 between the five authentic strains of Heterococcus group A (assigned to H. viridis , see text). In brackets, the total number of differences found with a certain molecular marker among the five strains. An asterisk marks the strain that is distinct from others by the presence of a “GCAA” indel in helix IV of ITS2. (DOCX 15 kb) [file 1471-2148-13-39-S7.docx]

|  |  | **rbcL (6)** | | |  |  | **psbA/rbcL (8)** | | | | | **ITS2 (2 + indel)** | | | | |
| --- | --- | --- | --- | --- | --- | --- | --- | --- | --- | --- | --- | --- | --- | --- | --- | --- |
|  |  | 1 | 2 | 3 | 4 |  | 1 | 2 | 3 | 4 |  | 1 | 2 | 3 | 4 |  |
| 1 | *H. viridis* SAG 835-3 |  |  |  |  |  |  |  |  |  |  |  |  |  |  |  |
| 2 | *H. mainxii* SAG835-6 | 3 |  |  |  |  | 1 |  |  |  |  | 0 |  |  |  |  |
| 3 | *H. marietanii* SAG 835-7 | 3 | 0 |  |  |  | 1 | 0 |  |  |  | 0 | 0 |  |  |  |
| 4 | *H. brevicellularis* SAG 835-1* | 4 | 5 | 5 |  |  | 7 | 6 | 6 |  |  | 2 | 2 | 2 |  |  |
| 5 | *H. moniliformis* SAG 835-8 | 4 | 5 | 5 | 0 |  | 4 | 3 | 3 | 5 |  | 2 | 2 | 2 | 0 |  |
|  |  |  |  |  |  |  |  |  |  |  |  |  |  |  |  |  |
